# 1560. Factors Driving Decisions in the use of HIV Pre-Exposure Prophylaxis: Data from a Real-World Study in the United States

**DOI:** 10.1093/ofid/ofad500.1395

**Published:** 2023-11-27

**Authors:** Yohance Whiteside, Abigail McMillan, Fritha Hennessy, Phoebe Salmon, Tim Holbrook, Bekana K Tadese

**Affiliations:** Merck & Co., Inc., Philadelphia, Pennsylvania; Adelphi Real World, Bollington, United Kingdom, Bollington, England, United Kingdom; Adelphi Real World, Bollington, United Kingdom, Bollington, England, United Kingdom; Adelphi Real World, Bollington, United Kingdom, Bollington, England, United Kingdom; Adelphi Real World, Bollington, United Kingdom, Bollington, England, United Kingdom; Merck & Co., Inc., Rahway, New Jersey, United States, North Wales, Pennsylvania

## Abstract

**Background:**

Although pre-exposure prophylaxis (PrEP) is recommended for people at risk of acquiring HIV, usage remains low. Our study used real-world data to characterise the factors driving decision-making in PrEP use, among current PrEP users (PU) and at-risk non-users (NU).

**Methods:**

Data were drawn from the Adelphi PrEP Disease Specific Programme (DSP)™, a real-world, cross-sectional survey of PU, NU, and physicians in the United States between December 2021 and June 2022. PU, NU, and physicians completed distinct questionnaires. Physicians (n=61) reported demographic data and PrEP usage for next 8 PU and 2 NU. These individuals were invited to complete a questionnaire reporting reasons for/against PrEP use (PU n=196; NU n=38).

**Results:**

Mean age [SD] of PU (n=480) and NU (n=121) was 35.3 [10.8] and 32.5 [10.8] years, respectively. Majority were male (PU 87%; NU 79%) and men who have sex with men (MSM) (PU 75%, NU 62%). Overall, 90% PU were taking PrEP daily and reported fear of contracting HIV (79%) and at-risk behaviours as major drivers for PrEP usage (Figure 1). Physicians reported that of the NU who chose not to start PrEP, 49% stated this was due to not wanting long-term medication (Figure 2). PrEP stigma was a concern for both PU (50%) and NU (65%).

**Figure:**
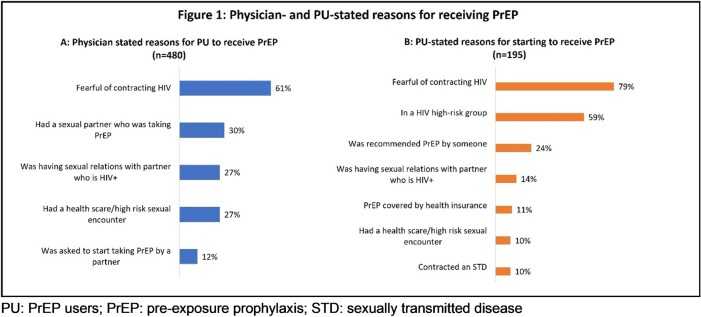

**Figure:**
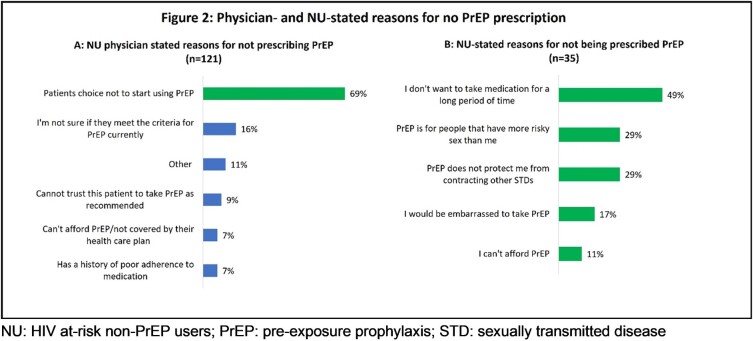

**Conclusion:**

This study provides data on unmet needs and potential drivers for PrEP uptake from the healthcare provider, PU and NU perspectives. Understanding factors that influence the decision to use PrEP may highlight the attributes needed in new products to increase uptake.

**Disclosures:**

**Yohance Whiteside, PhD, MSPH**, Merck & Co., Inc: Employee|Merck & Co., Inc: Stocks/Bonds **Bekana K. Tadese, PhD, MPH**, Merck and Co Inc: Employee

